# Supplementary material for: Borrelia burgdorferi Secretes c-di-AMP as an Extracellular Pathogen-Associated Molecular Pattern to Elicit Type I Interferon Responses in Mammalian Hosts
Source: bioRxiv. 2024 Aug 16:2024.08.13.607721. Preprint. [Version 1] doi: 10.1101/2024.08.13.607721 (PMC11343124; doi:10.1101/2024.08.13.607721)
Supplement: Supplement 1 [file NIHPP2024.08.13.607721v1-supplement-1.pdf]

bioRxiv preprint doi: https://doi.org/10.1101/2024.08.13.607721; this version posted August 16, 2024. The copyright holder for this preprint (which was not certified by peer review) is the author/funder, who has granted bioRxiv a license to display the preprint in perpetuity. It is made available under aCC-BY 4.0 International license.

SUPPLEMENTAL MATERIALS

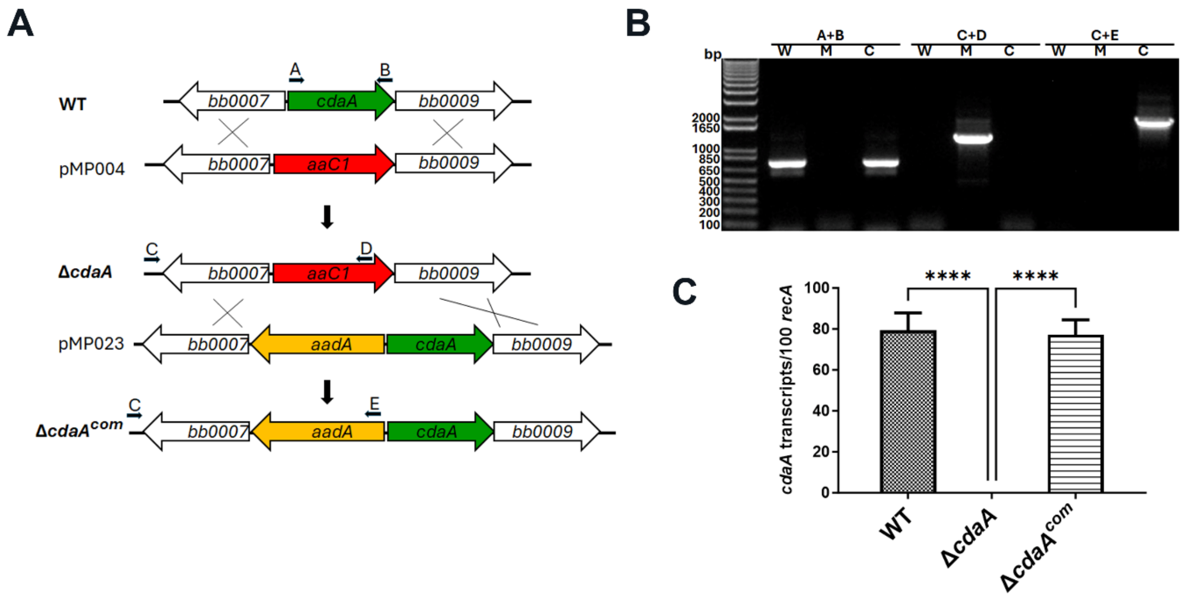

Supplemental Figure S1. Construction of the *cdaA* mutant. (**A**) Strategy for constructing the *cdaA* mutant (Δ*cdaA*) and the complemented strain (Δ*cdaA*^{com}). pMP004 and pMP023 are the suicide plasmids used for transformation and construction of the *cdaA* mutant and the complemented strain, respectively. Arrows with labels A-E indicate the positions of each primer for PCR analyses. (**B**) PCR analysis of wild-type (W), the *cdaA* mutant (M) and the Δ*cdaA* complemented strain (C) strains. The specific primer pairs used in PCR are indicated at the top. (**C**) qRT-PCR analysis of *cdaA* expression. The copy numbers of *cdaA* mRNA were normalized with 100 copies of a house-keeping gene *recA*. ***, $p < 0.0001$ using one-way ANOVA.

bioRxiv preprint doi: https://doi.org/10.1101/2024.08.13.607721; this version posted August 16, 2024. The copyright holder for this preprint (which was not certified by peer review) is the author/funder, who has granted bioRxiv a license to display the preprint in perpetuity. It is made available under aCC-BY 4.0 International license.

Supplemental Table S1: Primers for qRT-PCR and PCR used in this study

**PURPOSE**	**PRIMER SEQUENCE (5' to 3')**
qRT-PCR for IFNβ	F: GCCTTTGCCATCCAAGAGATGC<br>R: ACACTGTCTGCTGGTGGAGTTC
qRT-PCR for CXCL9	F: TTGGGCATCATCTTCCTGGAGCAG<br>R: GAGGTCTTTGAGGGATTTGTAGTGG
qRT-PCR for CXC10	F: CCTGCCCACGTGTTGAGAT<br>R: TGATGGTCTTAGATTCCGGATTC
qRT-PCR for CXCL11	F: AGGAAGGTCACAGCCATAGC<br>R: CGATCTCTGCCATTTTGACG
qRT-PCR for GBP2	F: CTACCGCACAGGCAAATCCTAC<br>R: GTCATTCTGGTTGTCACCCTTCTC
qRT-PCR for IFIT1	F: ATGGGAGAGAATGCTGATGGTG<br>R: TGTCAAGGAACTGGACCTGCTC
qRT-PCR for IFIT2	F: CAACGAGTAAGGAGTCACTGGAGAG<br>R: TTGCTGGATGAAGCCCTCAG
qRT-PCR for Actin	F: GTAACAATGCCATGTTCAAT<br>R: CTCCATCGTGGGCCGCTCTAG
Primer A in Fig. S1	CACCATGATAGACATAAATGATTTAAATC
Primer B in Fig. S1	TTACTCTATTAGCTCTAGATTTAAC
Primer C in Fig. S1	AACTCACATGCCCACACTTT
Primer D in Fig. S1	CAACAACCGCTTCTTGGTCG
Primer E in Fig. S1	ACTGCCATGGATGCATGCTCGAGCGGCCGCCAG

F, forward primer; R, reverse primer.
